# Endovascular Treatment of an Iatrogenic Inferior Mesenteric Arteriovenous Fistula Presenting as Massive Lower Gastrointestinal Hemorrhage: A Case Report

**DOI:** 10.1155/crra/3779245

**Published:** 2026-07-22

**Authors:** Maria Mihailescu, Clayton W. Commander, Robert Dixon, Nicole A. Keefe

**Affiliations:** ^1^ Department of Radiology, University of Pittsburgh Medical Center, Pittsburgh, Pennsylvania, USA, upmc.com; ^2^ Radiology Imaging Associates Ocala Division, Ocala, Florida, USA; ^3^ Department of Radiology, University of Arkansas for Medical Sciences, Little Rock, Arkansas, USA, uams.edu; ^4^ Department of Radiology, University of North Carolina at Chapel Hill, Chapel Hill, North Carolina, USA, unc.edu

**Keywords:** arteriovenous fistula, case report, coil embolization, embolization, inferior mesenteric artery

## Abstract

Inferior mesenteric artery arteriovenous fistulae are rare, acquired connections between arterial and venous systems associated with a range of symptoms, from abdominal pain to life‐threatening lower gastrointestinal hemorrhage. We report an iatrogenic inferior mesenteric artery arteriovenous fistula in a 30‐year‐old female, presenting with massive hematochezia 1 month after a left hemicolectomy. Angiography demonstrated a high‐flow vascular connection between the inferior mesenteric artery and rectal veins, which was treated with n‐butyl‐2‐cyanoacrylate mixed with ethiodized oil for arteriovenous connection occlusion followed by coil embolization of the feeding arteries. Follow‐up imaging at 8 months showed no recanalization of the fistula, and she remains asymptomatic 4 years later. Iatrogenic inferior mesenteric artery arteriovenous fistulae, though rare, should be considered after colorectal surgery as a possible cause of recurrent lower gastrointestinal hemorrhage, even within weeks of the operation; dilated mesenteric varices and premature venous filling on the arterial phase are key CT findings. Endovascular management targeting the arteriovenous communication and feeding arteries can achieve durable occlusion in these high‐flow fistulae.

## 1. Introduction

Inferior mesenteric artery arteriovenous fistulae (IMAVFs) are acquired high‐flow vascular connections between the arterial and venous systems that bypass capillary networks. By contrast, arteriovenous malformations (AVMs) are congenital and develop when undifferentiated embryonic vessels fail to regress, creating a nidus with multiple arterial feeders and fragile outflow veins [1]. Among approximately 200 reported splanchnic arteriovenous fistulae (AVFs) and AVMs, the majority arise from hepatic, splenic, or superior mesenteric vessels; the inferior mesenteric artery (IMA) is the least frequently involved [1]. IMAVFs most commonly occur following surgery but have been reported after trauma and associated with malignancy. Only 17 cases of IMAVFs have been reported in the literature [2–4]. We report a case of an IMAVF presenting with life‐threatening rectal hemorrhage 1 month after a left hemicolectomy, highlighting the preprocedure and postprocedure imaging findings and the emergent endovascular management.

## 2. Case Presentation

A 30‐year‐old female with a past medical history of hidradenitis suppurativa (HS) on high‐dose adalimumab and no personal or family history of inflammatory bowel disease (IBD) presented at an outside hospital with abdominal pain and frequent loose stools. Infectious workup was negative, and her inflammatory markers were mildly elevated. Outside hospital contrast‐enhanced CT demonstrated left‐sided colitis with mucosal congestion extending from the descending to the rectosigmoid colon. Biopsy was significant for focal active colitis and superimposed ischemic changes. She was treated empirically for bacterial colitis and discharged on a course of antibiotics after symptomatic improvement.

She represented with worsening symptoms and onset of bright red blood per rectum, raising concern for IBD. Corticosteroids were initiated without clinical improvement. Sigmoidoscopy showed findings consistent with ischemia, prompting a mesenteric angiogram at the outside hospital. The angiogram demonstrated occlusion of most of the IMA branches, and anticoagulation with heparin was initiated.

One week later, she decompensated with fever and tachycardia. She was transferred to our institution and ultimately required urgent left hemicolectomy with end transverse colostomy. Intraoperatively, the colon was firm and severely ischemic, with friable mesentery limiting visualization. A formal IMA ligation was not performed; however, IMA branches were oversewn during mesentery division to control bleeding. Surgical pathology showed extensive mucosal ischemic changes with small‐vessel intravascular thrombi. A hypercoagulable workup was unrevealing, and no definitive etiology for her ischemic colitis was identified. She was discharged 2 weeks later on a 3‐month course of apixaban.

**Figure 1:**
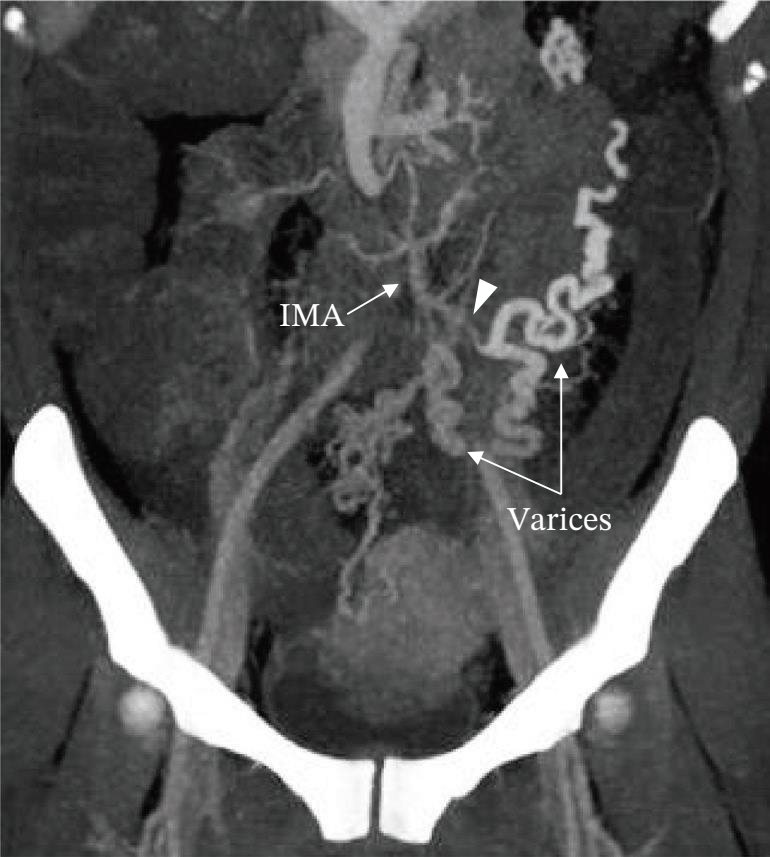
Coronal contrast‐enhanced CT obtained at an outside hospital 2 weeks preprocedure. Between the IMA and dilated mesenteric varices, a possible arteriovenous communication can be seen on retrospective review (arrowhead).

She presented again a month later at an outside hospital with profuse hematochezia and abdominal pain without bloody colostomy output. Portal venous phase contrast‐enhanced CT demonstrated thickening of the rectal stump with inflammatory changes. With knowledge of the final diagnosis, a retrospective review of this outside imaging, which was not available at the time of our procedure, showed dilated mesenteric varices with possible arteriovenous communication involving the IMA (Figure 1). The arteriovenous communication was not recognized prospectively, likely because bowel wall thickening from the IMAVF overlaps with that of other more common conditions higher on the differential. Rectal‐stump thickening could plausibly reflect persistent ischemic colitis of unknown origin that had prompted her initial presentation, arterial ischemia secondary to oversewn IMA branches during left hemicolectomy, or IBD that was considered earlier in the course; all three were raised as differential considerations at that time.

After 5 days, she was transferred to our hospital. On arrival, she was hypotensive, tachycardic, and profoundly anemic, but temporarily stabilized after transfusion. Sigmoidoscopy showed moderate inflammation with ulceration of the rectum, and her symptoms were attributed to proctitis. Five days later, she developed significant rectal bleeding. During a bedside sigmoidoscopy, inadvertent disruption of a large clot precipitated massive rectal hemorrhage, and she became hemodynamically unstable. She was intubated, the massive transfusion protocol was activated, and interventional radiology was consulted. Due to clinical urgency, no CT imaging was obtained, and she was taken directly to angiography.

**Figure 2:**
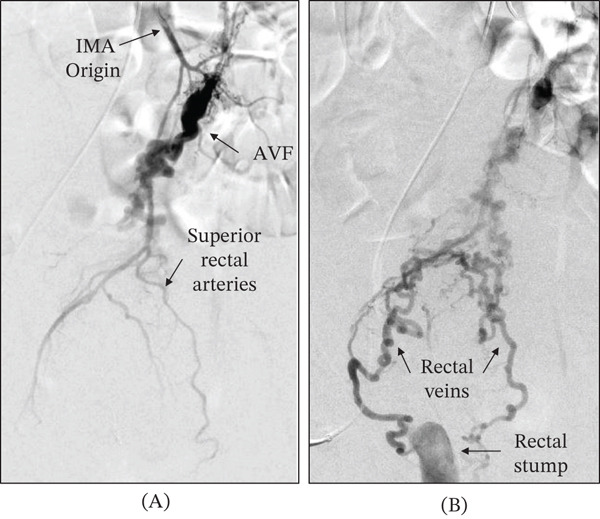
(A) Late arterial and (B) venous phase of IMA angiography. There is a large AVF connection measuring 1.0 cm between the IMA and rectal veins with contrast extravasation in the rectal stump.

Arterial access was obtained in the right common femoral artery, and thorough pelvic and mesenteric angiography was performed to locate the source of bleeding. The IMA was selectively catheterized using the 5 Fr Sos‐2 reverse curve catheter, and angiography revealed a large complex AVF between the IMA and rectal veins, with tortuous varices measuring 0.5–1 cm and active extravasation into the rectal stump (Figure 2). A Progreat microcatheter was placed through the base catheter and advanced over a wire into the IMA. Subselective angiography was performed, identifying the descending branch of the left colic artery and a sigmoid branch as dominant feeding arteries to the AVF.

**Figure 3:**
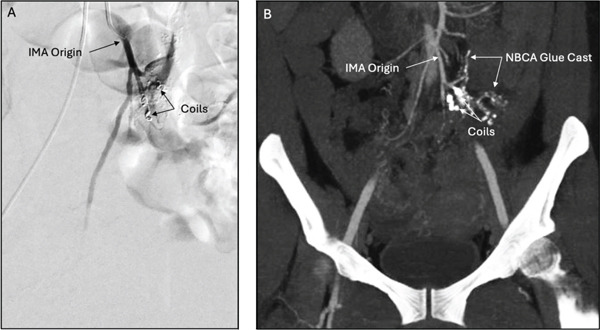
Postembolization imaging. (A) Final IMA angiography demonstrates coils within the sigmoid and descending left colic branches with resolution of flow through the AVF and no active extravasation. (B) Coronal CT angiography at 8 months postprocedure confirms complete occlusion of the IMAVF. Coils and NBCA glue cast are identified without evidence of recanalization.

The microcatheter was advanced into the descending branch of the left colic artery near the arteriovenous connection, and 0.5 mL of n‐butyl‐2‐cyanoacrylate (NBCA) mixed with ethiodized oil (6:1 ratio) was injected to occlude the fistulous connection and proximal venous outflow. Repeat angiography demonstrated reduced but persistent flow. The feeding descending left colic branch was embolized with a single 3‐to‐2‐mm tapered, 2‐cm Tornado pushable microcoil (Cook Medical, Bloomington, Indiana). The microcatheter was then used to select the contributing sigmoid branch, and this was embolized with two 3‐to‐2‐mm tapered, 2‐cm Tornado pushable coils. Final angiography confirmed markedly diminished flow; technical success, defined as no residual extravasation, was achieved (Figure 3). All catheters were removed, and the right common femoral artery access site was closed with Angio‐Seal.

She received a total of 12 units of packed red blood cells, 12 units of fresh frozen plasma, and two units of platelets prior to embolization. Postprocedure, she had no further hematochezia. The next morning, she no longer required pressors, and her hemoglobin remained stable without further blood products. She underwent colostomy takedown 5 months later. Follow‐up CT angiography at 8 months demonstrated complete occlusion of the IMAVF, defined as no contrast opacification beyond the coils and glue, and no imaging evidence of collateral recruitment (Figure 3). At her last follow‐up visit, 4 years postembolization, she remained asymptomatic with no clinical evidence of recurrence. She was thankful that the cause of her symptoms was eventually identified and successfully treated. A timeline of clinical events is summarized in Table 1.

**Table 1:** Timeline of clinical events.

Timepoint	Event	Clinical details
Day 0	Initial presentation with abdominal pain and loose stools	CT showed left‐sided colitis
Days 9–15	Clinical deterioration with bright red blood per rectum	Mesenteric angiogram demonstrated occlusion of most of the IMA branches
Weeks 3–5	Clinical decompensation, urgent left hemicolectomy with end transverse colostomy	IMA branch vessels were oversewn during mesenteric division to control bleeding
Week 9	Presented with profuse hematochezia and abdominal pain, emergent angiography and embolization of IMA‐rectal veins AVF	Large AVF between IMA and rectal veins with active extravasation into the rectal stump seen on angiography, embolized with NBCA/ethiodized oil injected at the AVF bed and coil embolization of feeding arteries
5 months postembolization	Colostomy takedown	No further episodes of hematochezia in the interim
8 months postembolization	Follow‐up	CT angiography demonstrated durable occlusion of the IMAVF
4 years postembolization	Last follow‐up	She remains asymptomatic

Abbreviations: AVF, arteriovenous fistula; IMA, inferior mesenteric artery; NBCA, n‐butyl‐2‐cyanoacrylate.

## 3. Discussion

IMAVFs are exceedingly rare, with fewer than 20 cases reported in the literature [2–4]. They most often result from surgery, thought to arise from a transfixation suture passing through both an artery and a vein, or from simultaneous ligation of adjacent vessels [2]. IMAVF secondary to surgery or trauma may present within weeks, as in this case, or up to 37 years later, with most cases occurring years to decades after the causative injury or procedure [5, 6].

The cause of her IMA thrombosis and resulting colonic ischemia remained unclear. The patient had no known risk factors for mesenteric ischemia, and a hypercoagulable workup was negative. Although no etiology was identified, she was on high‐dose adalimumab for HS, and TNF‐α inhibitors have been associated with thrombotic events on pharmacovigilance analysis [7]. This association offers a possible explanation; however, no causal relationship between adalimumab and thrombosis has been established.

Our patient′s vascular lesion was considered an IMAVF rather than an AVM because no arteriovenous shunt was seen on mesenteric angiogram performed at the outside hospital prior to the left hemicolectomy; a congenital AVM would have been angiographically evident on that study. Furthermore, the fistula involved the descending left colic and sigmoid branches, vessels that were oversewn during the left hemicolectomy mesenteric division when a suture could have inadvertently transfixed an adjacent artery and vein. With no history of trauma or regional malignancy, an iatrogenic origin is most likely.

The diagnosis of IMAVF or AVM can be delayed because their clinical and imaging features can mimic other common conditions [1, 8]. In our patient, rectal bleeding and abdominal pain, with inflammation and bowel wall thickening of the rectum on imaging, were attributed to ischemic colitis or IBD. In retrospect with knowledge of the final diagnosis, portal venous phase CT obtained 2 weeks prior to embolization displayed dilated mesenteric varices and a possible arteriovenous communication, clues to an IMAVF. These findings would have been even more conspicuous on arterial‐phase imaging, manifesting as early opacification of the draining veins and dilated feeding arteries.

IMAVFs and AVMs can be treated surgically or endovascularly, and the approach depends on the patient′s hemodynamic status and feasibility of intervention. Surgical resection of these vascular lesions has been successful without major complications in every case reported in the literature, and offers a salvage option when endovascular management fails [9]. However, if a patient is too hemodynamically unstable to tolerate surgery, as in our case, endovascular management may be the only option.

Angioarchitecture is the key consideration when planning embolization strategy and assessing complication risk [9]. Simple fistulae with few feeder vessels, typically seen in IMAVFs, are effectively treated with coil embolization of arterial feeders, occasionally combined with liquid embolics to occlude the AVF junction. For large‐caliber feeders at risk for coil migration, oversized Amplatzer plugs (Abbott, Chicago, Illinois) can be deployed in the IMA or other proximal inflow vessels [10].

Inferior mesenteric artery arteriovenous malformations (IMAVMs) require liquid embolics for nidus occlusion. When there are multiple arterial feeders and a dominant venous outflow tract, a venous approach via percutaneous transhepatic access or direct puncture of the venous varix has been reported [11, 12]. A complex IMAVM was successfully treated via transhepatic venous retrograde injection of sodium tetradecyl sulfate foam through an occlusion balloon followed by coil embolization [12]. Coil embolization of the AVM outflow tract via transhepatic access followed by transarterial liquid embolization of the nidus is another viable approach [13]. When small branch vessels arise from the target arterial inflow to the AVM, protective coiling of these branches prior to injection of the liquid embolic is essential to prevent nontargeted embolization [8].

The IMAVF in our case was complex, with two arterial feeders and a conglomerate of rectal veins. A permanent liquid embolic was necessary to penetrate the multiple arteriovenous connections, eliminate the pressure gradient driving the shunt, and reduce the risk of collateral recruitment that can occur if only the inflow arterial vessels are treated [2]. NBCA/ethiodized oil was selected for its rapid polymerization, which was critical given our patient′s tenuous hemodynamic status. Furthermore, the NBCA/ethiodized oil ratio was tailored to control viscosity and polymerization time based on fistula flow and anatomy. The primary risks of NBCA/ethiodized oil in this case were catheter adhesion and nontargeted embolization via passage into the portal venous system; the latter can be mitigated by meticulous microcatheter positioning, controlled injection, and an NBCA/ethiodized oil ratio appropriate for the blood flow rate.

Other liquid embolic agents described in the IMAVF and AVM literature were not preferred in this context. Onyx requires slow injection to precipitate, with potential precoiling to reduce shunt flow, limiting its use in emergent high‐flow fistula occlusion. Similarly, sodium tetradecyl sulfate requires relatively prolonged vessel wall contact for endothelial sclerosis. Although this could be facilitated with initial flow reduction, it was not feasible in an emergent setting. Emerging embolic agents such as Lobo Vascular Occluder (Okami Medical, Aliso Viejo, California) or liquids such as Obsidio (Boston Scientific, Marlborough, Massachusetts) may also function in these lesions.

Following arteriovenous occlusion with NBCA/ethiodized oil, the feeding arteries were embolized with coils to reduce residual inflow and prevent collateral recruitment. Coils, rather than additional NBCA, were chosen to decrease the risk of nontarget embolization. Residual inflow in IMAVF leads to recurrence or persistence via collateral recruitment, which may require repeat embolization [2]. In AVMs, partial thrombosis of the nidus with residual inflow can have more serious consequences. In one reported case, increased intranidal pressure led to rupture of fragile varices, requiring emergent surgery [14].

This report has several limitations. The arteriovenous communication on preprocedure CT was appreciated only on retrospective review with knowledge of the final diagnosis and therefore may be more apparent in hindsight than it would have been prospectively. A second limitation is that fistula etiology is inferred to be iatrogenic based on its absence on preoperative angiography, the anatomic correspondence to the area of IMA division, and its temporal relationship with surgery; however, there is no histopathologic confirmation. Regarding the intervention, the emergent clinical setting limited the opportunity to compare alternative embolization approaches during the procedure. Finally, this is a single case, and the findings cannot be generalized, especially since the management of IMAVF is highly varied in the literature.

## 4. Conclusion

Iatrogenic IMAVF should be considered in the differential diagnosis for bowel ischemia or unexplained recurrent lower gastrointestinal hemorrhage after colorectal surgery, even within weeks of the operation. Bowel wall thickening with adjacent inflammatory changes seen on imaging is nonspecific and may be interpreted as recurrent ischemic colitis or IBD, as was the case in our patient. Premature filling of draining veins on arterial‐phase CT and dilated mesenteric varices are key findings that point to an underlying IMAVF, and dedicated arterial‐phase imaging can make these findings more apparent when a fistula is suspected. Once identified, high‐flow visceral AVFs can be treated with a combined strategy targeting the arteriovenous connection followed by embolization of the feeding arteries, achieving durable results by preventing collateral recruitment and recurrence. Our patient′s IMAVF was successfully embolized using NBCA/ethiodized oil for arteriovenous connection occlusion followed by coil placement to reduce residual inflow. In this patient, endovascular management was curative in a life‐threatening situation when surgery was not feasible, with sustained occlusion 4 years later.

## Funding

No funding was received for this manuscript.

## Ethics Statement

Institutional review board (IRB) approval was not required for this single‐patient case report in accordance with University of Pittsburgh Medical Center policy. Written consent for publication was obtained from the individual person′s data included in this study.

## Conflicts of Interest

The authors declare no conflicts of interest.

## Data Availability

Data sharing is not applicable—no new data generated, or the article describes entirely theoretical research. This case report was prepared in accordance with CAse REport (CARE) guidelines.
